# The relationship between glycine levels in collagen in the anterior rectus sheath tissue and the onset of indirect inguinal hernia: A cross-sectional study

**DOI:** 10.1016/j.amsu.2021.103166

**Published:** 2021-12-09

**Authors:** Amir Fajar, Warsinggih, Erwin Syarifuddin, Joko Hendarto, Ibrahim Labeda, Ronald Erasio Lusikooy, Mappincara, Muhammad Iwan Dani, Samuel Sampetoding, Muhammad Ihwan Kusuma, Julianus Aboyaman Uwuratuw, Muhammad Faruk

**Affiliations:** a Division of Digestive, Department of Surgery, Faculty of Medicine, Hasanuddin University, Makassar, Indonesia; b Division of Digestive, Department of Surgery, Dr. Wahidin Sudirohusodo General Hospital, Makassar, Indonesia; c Department of Biostatistics, Faculty of Public Health, Hasanuddin University, Makassar, Indonesia; d Department of Surgery, Faculty of Medicine, Hasanuddin University, Makassar, Indonesia

**Keywords:** Inguinal hernia, Glycine, Collagen, ELISA assay, Cross-sectional study

## Abstract

**Introduction:**

An inguinal hernia is a protrusion of abdominal-cavity contents through the inguinal canal. Protection against an inguinal hernia depends on the integrity of fascial tissue, which is maintained by collagen. Collagen is a structural protein consisting of amino acids, the most common of which is glycine. This study aimed to determine the relationship between glycine and the appearance of lateral inguinal hernias. To this end, the researchers examined the profile of collagen and glycine levels in the tissue of the sheath of the rectus femoris in patients with lateral inguinal hernia (indirect inguinal hernia).

**Methods:**

The study used a cross-sectional design to determine glycine levels in rectus anterior sheath tissue in patients with indirect inguinal hernia. Examination of collagen glycine levels was conducted using the ELISA (Enzyme-Linked immunosorbent assay) method. Data were analyzed using the Statistical Package for the Social Science (SPSS) program. An ANOVA test, Pearson's correlation test, and Spearman's correlation test were also performed. A p-value <0.05 was said to be significant.

**Results:**

Across 72 samples, the mean body mass index (BMI) was 22.5 kg/m2 and, the mean clinical onset was 28.02 months. Correlation tests showed a correlation between glycine levels and clinical onset (p = 0.026). The ANOVA test showed a difference between glycine levels with age group (p = 0.025) and BMI (p = 0.015). The correlation between glycine levels and clinical-grade (p = 0.416) was not statistically significant.

**Conclusion:**

There is a significant relationship between glycine levels and age, BMI, and clinical onset of indirect inguinal hernia.

## Highlights

• The stability of the abdominal wall depends on the integrity of muscle and fascial tissue.
• Collagen is a structural protein that helps tissues maintain their mechanical characteristics, structure, and shape.
• The collagen structure contains amino acids such as glycine, proline, hydroxyproline, alanine, and hydroxylysine.
• The high glycine content of collagen is vital to promoting collagen turnover, as its deficiency reduces collagen turnover.
• There is a relationship between glycine levels and age, body mass index, and clinical onset of indirect inguinal hernia.

## 1 Introduction

An inguinal hernia is a protrusion of the contents of the abdominal cavity through the inguinal canal due to a defect in the abdominal wall [1,2]. The stability of the abdominal wall depends on the integrity of muscle and fascial tissue. Collagen is a structural protein that helps to maintain the mechanical characteristics, structure, and shape of tissues. Moreover, collagen is a significant component of the extracellular matrix and has good resistance to support structures such as the fascia [3]. Type I collagen dominates the fascia, where this structure plays a significant role in the incidence of inguinal hernia [4]. A previous study reported that the ratio of type I and type III collagen was significantly decreased in patients with inguinal hernia. A disturbance in the ratio between collagen types I and III contributed to tissue weakness in fascial tissue or aponeurosis [5,6].

Another study found that in patients with direct inguinal hernia, the anterior rectus sheath above the defect was thinner than normal [4]. There was a decrease in the proliferation of fibroblasts and collagen and hydroxyproline content was as high as 19.2% [2,7,8]. Studies on the transverse fascia of patients with hernias have also found abnormalities in the collagen content. The extensibility and elasticity of the transverse fascia were also significantly increased in patients with direct hernias [9]. A further study found a 23.7% lack of type I collagen in patients with indirect inguinal hernia transverse fascia. Decreases in collagen and elastin have also been observed in the rectus sheath and peritoneal cavity of patients with direct and indirect inguinal hernias [10]. Indirect inguinal hernia were due to the vaginal patenting process itself, but also to abnormalities in the structure of the inner ring, disruption of the transversal fascia, and abnormal muscle function [11].

The collagen structure contains amino acids such as glycine, proline, hydroxyproline, alanine, and hydroxylysine. Glycine is the simplest of the amino acids, including non-essential amino acids, and is one of the major structural units of collagen, absorbing approximately 30% of the amino acids in collagen [12]. Moreover, glycine is required to wrap around the three alpha chains of the tropocollagen molecule [13,14]. The high glycine content of collagen suggests the importance of the availability of these amino acids in promoting healthy collagen turnover, as its deficiency reduces the turnover of proteins, especially collagen. Glycine produced from the breakdown of collagen and the procollagen cycle must be recycled to meet the body's needs for glycine [15]. The study found that glycine supplementation caused quantitatively higher collagen expression than the control group. In vitro studies, meanwhile, have shown that an adequate glycine intake is important for collagen synthesis and maximum growth performance. Glycine is directly linked to the organization and remodeling of collagen fibers [16]. Adequate availability of glycine promotes optimal collagen synthesis, maximum growth, and optimal health [12].

Developing a deep understanding of the pathogenesis of inguinal hernia is crucial to promoting better outcomes for those who suffer from the condition. To this end, research must attempt to identify the existence of a relationship between collagen abnormalities and the development of inguinal hernias, and to study the importance of glycine in supporting optimal collagen synthesis and function to promote the creation of optimal health. This aim has prompted us to examine the profile of glycine collagen levels in rectus anterior sheath tissue in patients with lateral inguinal hernia (indirect inguinal hernia).

## 2 Methods

This observational analytical study uses a cross-sectional study to determine the profile of glycine collagen levels in rectus anterior sheath tissue in patients with indirect inguinal hernia. We conducted this research at Dr. Wahidin Sudirohusodo Hospital and Hasanuddin University Hospital, Makassar, Indonesia.

We obtained ethical approval for this research from the Ethics Commission, Faculty of Medicine, Hasanuddin University, number: 523/UN4.6.4.5.31/PP36/2021 and it has been registered with the Research Registry (no. 7361). Our work in this report is presented according to the Strengthening the Reporting of Cohort Studies in Surgery guidelines [17].

The inclusion criteria were patients with an indirect inguinal hernia who had not undergone surgery, patients who were prepared to participate in the research, and patients who had given their informed consent. Exclusion criteria were patients with an indirect inguinal hernia who had undergone surgery (relapse), patients who had a prostate disease or inguinal tumors, patients with chronic lung disease, and patients with a BMI of <18, 5 kg/m2 (undernutrition).

### 2.1 Inguinal hernias

Inguinal hernias are generally classified as direct, indirect, or femoral based upon the site of herniation relative to surrounding structures [[18], [19], [20]]. The study population consisted of all the patients who were treated for indirect inguinal hernia at our institution. The sample for this study is part of the total research population that meets the inclusion and exclusion criteria (consecutive sampling).

### 2.2 Body mass index

The body mass index (BMI) classification we used is based on the BMI classification of the Indonesian Ministry of Health: underweight (<18.5 kg/m^2^), normal weight (≥18.5 – <24.9 kg/m^2^), overweight (≥25.0 – <27 kg/m^2^), and obese (27.0 kg/m^2^).

### 2.3 Age groups

We also established several age groups according to the guidelines laid out by the Indonesian Ministry of Health (2009), which are as follows: youth age (12–25 years), young age (26–45 years), middle-aged (46–65 years), and elderly age (>65 years old).

### 2.4 Clinical onset

The clinical onset is the time span that starts when patients with indirect inguinal hernia begin to experience initial complaints related to the hernia diagnosis up until definitive management (surgery) has been carried out.

## 3 Sample extraction from the anterior rectus sheath

We removed the defect in the muscle tissue of the rectus femoris sheath during surgery with a size of 0.5 × 1 cm. The tissue was then washed with PBS buffer (pH 7.4) and weighed, before being homogenized in PBS solution by vortexing. The finished solution was stored at −200C. The solution should be centrifuged again at a 2000–3000 RPM speed for 15–20 min before proceeding with the examination.

### 3.1 Sample examination

According to the manufacturer's instructions, the examination of collagen glycine levels using the Enzyme-Linked immunosorbent assay kit (catalog no. EA7867Hu) reagents from the Bioassay Technology Laboratory (Shanghai, China) [21].

### 3.2 Statistical analysis

The data collected were analyzed using the SPSS Statistics 23.0 (IBM Corp. Released 2015. IBM SPSS Statistics for Windows, Version 23.0. Armonk, NY: IBM Corp.). A data normality test was performed using the Kolmogorov–Smirnov test. The correlation test was performed using the Pearson correlation test if the data distribution was normal, while the Spearman correlation test was used if the data distribution was not normal. P-values less than 0.05 were considered statistically significant. If there were several statistically significant variables from the results of the bivariate analysis, the multivariate analysis was continued using several linear tests.

## 4 Results

The study was conducted on 72 subjects, each of whom met the criteria (Table 1). Table 2 shows that the majority (87.55%) of the indirect inguinal hernia patients in this study fell into either the adult or elderly categories. The subjects’ BMIs, meanwhile, were mostly in the normal category, i.e., 64 out of 72 subjects (88.9%). Based on the appearance of a hernia, we found that the fastest clinical onset of hernia patients seeking treatment was 3 months, and the most extended onset was 90 months (7 years and 6 months).

**Table 1:** Characteristics of subjects.

	N	Mean	Std. deviation	Minimum	Maximum
Age (years)	72	45.18	11.10	19.00	70.00
BMI (kg/m^2^)	72	22.50	1.70	18.70	27.60
Clinical onset (months)	72	28.01	17.97	3.00	90.00
Glysine levels (pg/mL)	72	0.53	0.15	0.18	0.87

**Table 2:** Characteristics of subjects with inguinal hernia according to age and BMI.

Characteristics of subjects	n	(%)
**Age (years)**		
12–25	4	5.6
26–45	31	43.1
46–65	32	44.4
>65	5	6.9
**BMI (kg/m**^**2**^**)**		
normal weight (≥18.5 – <24.9)	64	88.8
overweight (≥25 – <27)	4	5.6
obesity (≥27)	4	5.6

**Fig. 1:**
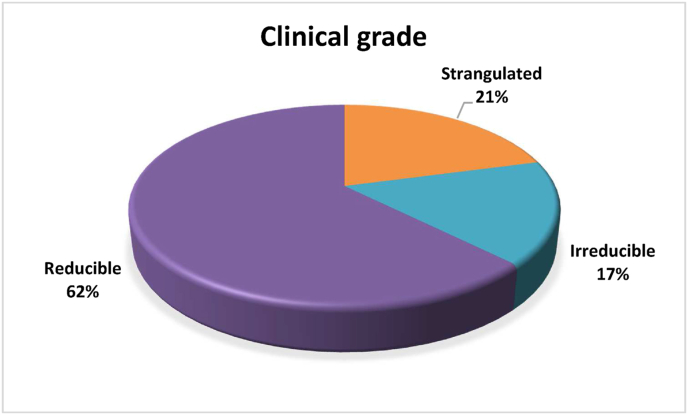
Patient characteristics according to clinical grade.

Based on clinical-grade checks performed on patients before they underwent surgery, we found that 45 out of 72 patients had a reducible hernia (62.0%), 12 patients had an irreducible hernia (17.0%), and 15 patients had a strangulated hernia (21.0%) (Fig. 1).

To assess the mean difference between glycine levels as a function of age, the ANOVA test was performed for each age group, obtaining a p-value = 0.025 (p < 0.05). It was therefore concluded that there was a statistically significant difference in mean glycine between age and glycine levels, although more generally, it was observed that the mean glycine content decreased with age (Table 3).

**Table 3:** Differences in average glycine levels by age.

Glycine levels						
Age	N	Mean	SD	Minimum	Maximum	p-value
Youth age	4	0.59	0.18	0.47	0.87	0.025
Young age	31	0.59	0.15	0.18	0.86	
Middle age	32	0.50	0.14	0.26	0.85	
Elderly age	5	0.40	0.04	0.36	0.46	

Table 4 shows the results of the post-hoc test using the LSD method. The results indicate that the mean glycine levels in the adult patient group had a statistically significant difference compared to the elderly group with p = 0.026 and the elderly group with p = 0.012.

**Table 4:** Post-hoc test using LSD method for glycine levels in the age group.

Group	Group	p-value
Young age	Youth age	0.907
	Middle age	0.026
	Elderly age	0.012
Elderly age	Youth age	0.057
	Young age	0.012
	Middle age	0.172

The ANOVA test was performed to assess the mean difference in glycine levels in each BMI group and obtained a p-value = 0.015 (p < 0.05). It was therefore concluded that there was a statistically significant mean difference between BMI and glycine levels. Plus, more generally, the average level of glycine increased depending on the BMI category (Table 5).

**Table 5:** Differences in average glycine levels according to BMI.

Glycine levels						
BMI	N	Mean	SD	Minimum	Maximum	p-value
Normal weight	64	0.5269	0.15046	0.18	0.87	0.015
Overweight	4	0.5331	0.09671	0.39	0.60	
Obesity	4	0.7551	0.15568	0.52	0.86	
Total	72	0.5399	0.15580	0.18	0.87	

Table 6 shows the results of the post-hoc test using the LSD method. The results indicate that the mean difference in glycine levels in the obese patient group has a statistically significant mean difference from the normal BMI group with a correlation value of p = 0.004 and in the overweight group p = 0.038.

**Table 6:** Post-hoc test of the LSD method of glycine levels on the BMI.

Group	Group	p-value
Obese	Normal weight	0.004
	Overweight	0.038

To assess the correlation between the onset of indirect inguinal hernia incidence and glycine levels, a Spearman's test was performed, with a p-value = 0.026 (p < 0.05) being obtained. It was therefore concluded that there was a statistically significant correlation between the appearance of the onset of a hernia and glycine levels with a correlation coefficient of −27.8% (low significance). This meant there was an inverse correlation whereby the longer the onset of the indirect inguinal hernia, the lower the glycine level (Table 7).

**Table 7:** Correlation between the onset of a hernia and glycine levels.

			Onset
**Spearman's**	**Glycine levels**	**Correlation Coeff (r)**	−0.263
		*p*	0.026
		n	72

In Table 8, we can see that the mean difference is based on clinical-grade and glycine levels; the ANOVA test was performed to assess the value of the difference in mean glycine levels in each clinical-grade group. The p-value = 0.416 (p > 0.05); therefore, it was concluded that there was no statistically significant difference between clinical-grade and glycine levels. More generally, we observed that average glycine levels appear to be at their lowest in the strangulated group.

**Table 8:** Differences in mean glycine levels according to clinical grade.

Glycine levels						
Clinical grade	N	Mean	SD	Minimum	Maximum	p-value
Strangulated	15	0.5008	0.16101	0.31	0.87	0.416
Irreducible	12	0.5195	0.14202	0.27	0.76	
Reducible	45	0.5584	0.15777	0.18	0.86	

To assess the relationship of all significant variables with glycine levels, multiple linear regression tests were performed. Based on the order of importance of variables on glycine levels, we found that the BMI group variable had the highest b-value of 0.710, followed by the age group variable (b = −0.590), and then the onset of indirect inguinal hernia (b = −0.001). The constant value was 0.643 with Sig 0.000 (Table 9).

**Table 9:** Multivariate analysis (multiple linear regression) between all significant variables with glycine levels.

Variable	Standard coefficient		Sig
	b-value	Std. error	
Constant	0.643	0.082	0.000
BMI	0.071	0.025	0.002
Age	- 0.059	0.035	0.044
Clinical onset	- 0.001	0.001	0.177

## 5 Discussion

A hernia is a condition that is triggered by both endogenous and exogenous factors [22]. Increased collagen degradation is also associated with the formation of a hernia [22,23]. The composition of collagen appears to affect changes in fascial and systemic tissues. Changes in collagen are also observed in patients with hernial recurrence; the amino acids that make up the main component of collagen are glycine, hydroxyproline, proline, and alanine [2,22].

Of the indirect inguinal hernia patients who participated in the study, the average age was 45.1 years, with the youngest participant being 19 years old and the oldest being 70 years old. It is estimated that more than 20 million inguinal hernia repairs are performed each year worldwide. Inguinal hernias occur in 75% of all abdominal wall hernias. The incidence of inguinal hernias has a bimodal distribution, peaking at around age 5 and after age 70, with males accounting for approximately 90% of all inguinal hernias and females accounting for approximately 10% [8,24].

The cumulative prevalence of inguinal hernia in men aged 25 to 34 was 5%, increasing to 10% for those aged 35–44 years, 18% for those aged 45–54 years, 24% for those aged 55–64 years, 31% for those aged 65–74 years, and 45% for those who were 75 years and over [25].

With regards to the BMI data of the hernia patients, we found that the lowest BMI was 18.7 kg/m2 and the highest BMI was 27.6 kg/m2, with the mean BMI being 22.5 kg/m2. A study by Rekha et al. concluded that a normal BMI was associated with a high incidence of inguinal hernia among the sexes. More specifically, a normal BMI in men was shown to indicate a greater likelihood of an inguinal hernia than was indicated by a low BMI in men. It has been widely hypothesized that obesity, owing to the fact that it causes increased abdominal pressure, is responsible for the increased frequency of inguinal hernias—but these results undermine such a hypothesis [26].

Indeed, the risk of developing an inguinal hernia has actually been reported to decrease in overweight or obese people in most studies. Rosemar et al., for instance, showed that in male patients aged 47–55 years with inguinal hernia, an increase in BMI of one unit (from 3 to 4 kg) reduced the risk of hernia by 4% [26]. However, based on the results of a study by Sorensen et al., the biological effects of collagen and glycine levels on age and gender factors continue to be debated [27]. Still, the clinical evidence for the incidence of inguinal hernias is highest in men, and the risk has been shown to increase with age [28]. According to a prospective cohort study conducted by Ruhl and Everhart, a high BMI is a protective factor against the risk of hernia [29]. Another study found a higher incidence of hernia in patients with a normal BMI compared to patients who were overweight or obese [26]. In this study, there was a statistically significant relationship between age and glycine levels. The mean ANOVA test obtained a p-value = 0.025, suggesting an inverse relationship in which the older a patient is, the higher their glycine level. Glycine is a non-essential amino acid synthesized from glutamate in a two-step pathway that requires NADPH. An experimental study in aging mice found that glycine, alanine, serine, tyrosine, and methionine decreased in plasma with aging. Taniguchi et al. reported that changes in structure and collagen levels were strongly influenced by age [30], while in a large-scale study involving a sample of children, they reported that collagen type I levels increased with age in line with glycine levels and other types of mature collagen [31]. A retrospective cohort study reported that significant risk factors for the incidence of hernias were age, smoking, and diabetes [26].

In this study, a statistically significant relationship was found between BMI and glycine levels. This relationship is based on the result of the mean ANOVA test between BMI and glycine levels (p-value = 0.025), which suggests a directly proportional relationship whereby the higher the BMI, the higher the glycine level. In a previous study, Serralde-Zuniga et al. assessed the amino acid profile of overweight and obese subjects, classifying the population according to their BMI as normal weight (20 to <25 kg/m2), overweight (25 to <30 kg/m2), and obese (30 kg)/m2). There was a lower incidence of hernia in overweight or obese young adults than in young adults of normal weight [32]. Sorensen et al. concluded that no significant evidence linked dietary factors in obese samples to collagen and glycine levels. Still, clinical obesity was reported as a predictor of recurrent hernia [27].

Kjaer et al. (2020) reported that malnutrition was associated with high infection rates and reduced resistance of local tissues in the transverse fascia. Therefore, the administration of multi-nutrient supplements in the control group was reported to increase collagen synthesis [33]. Another study reported that a high BMI is a protective factor against the incidence of hernias, whilst also showing that other factors, such as a cough, constipation, prostatism, and physical activity involving heavy weights, increase the risk of hernia [26,34].

In this study, a statistically significant relationship was found between the clinical onset of indirect inguinal hernia and glycine levels with a correlation coefficient of −26.3% (low significance), which means that there was an inverse relationship whereby the longer the onset of the hernia, the lower the glycine level. Consistent with findings reported by Taniguchi et al. [30], we also found that changes in tissue structure in adult hernia patients were linked to the secondary pathogenesis of collagen metabolism, which ultimately affects the levels of collagen and glycine in the hernias supporting tissues of the inguinal sheath. Indeed, collagen metabolism plays an important role in the incidence of hernias, whereby the primary pathogenesis is related to the onset of permeability of the vaginal process, which triggers the incidence of indirect inguinal hernias.

This study found no statistically significant relationship between clinical grade and glycine levels. Consistent with the previous study, in patients with direct inguinal hernia, the anterior rectus sheath over the defect was thinner than is typical due to decreased collagen, which in turn may be due to a decrease in fibroblast proliferation, as indicated by cultures of fibroblasts taken from the rectus femoris sheath [35]. The anterior rectus sheath of patients with direct inguinal hernias was thinner and had significantly lower collagen and elastin content and a decrease in the ratio of hydroxyproline to proline in patients with an indirect inguinal hernia with controls. These results suggest that patients with direct inguinal hernia have a thinner straight sheath with less collagen and a transverse fascia with higher levels of immature collagen and, therefore, they suffer the loss of fascial strength [10].

Stabnikov (2016) reported that the pathogenesis of hernia is the result of impaired collagen synthesis and amino acid deficiency, where amino acids form collagen, and the main amino acids that form collagen are glycine. This amino acid is a component that affects the quality of collagen [36].

After multivariate analysis, we found that BMI had the strongest influence on glycine levels in patients with lateral inguinal hernia, with age group being the next most influential variable. In contrast, the clinical onset had a minimal effect on glycine levels. This is in line with research findings by Serralde-Zuniga et al., who concluded that a person with a BMI in the obese category has a more complex amino acid profile than a person with a BMI in the underweight category because we know that glycine is a non-essential amino acid [32]. Furthermore, according to Harrison et al. [10], malnutrition is an exogenous factor, while age is an endogenous factor. It is also a factor that affects the formation of fibroblasts, which in turn affects collagen metabolism, which is considered one of the factors that leads to the onset of inguinal hernia. Moreover, Asserin et al. demonstrated that the collagen content in the body can be affected by the aging process and poor diet [37].

While this study produced some notable findings, it also had some limitations. Firstly, it utilized a small sample size, and its duration was relatively short. Thus, there is a need for further research with a larger sample size and longer study duration. Secondly, the study focused largely on glycine levels. Third, confounding factors that could not be controlled by convenient sampling include the co-occurrence of other factors such as smoking, co-morbidities, and lifestyle. Based on our study results, we recommend more large-scale studies and examine different ELISA levels such as proline, hydroxyproline, alanine, and hydroxylysine in the collagen of rectus sheath tissue in patients with indirect inguinal hernia.

## 6 Conclusion

There is a significant relationship between glycine levels and age, BMI, and the clinical onset of indirect inguinal hernia. However, there is no relationship between glycine levels and the clinical grade of indirect inguinal hernia.

## Ethical approval

All procedure for human experiment has been approved by Ethics Commission Faculty of Medicine, Hasanuddin University number: 523/UN4.6.4.5.31/PP36/2021.

## Sources of funding

None.

## Author contribution

All authors made a significant contribution to the work reported, whether that is in the conception, study design, execution, acquisition of data, analysis and interpretation, or in all these areas; took part in drafting, revising or critically reviewing the article; gave final approval of the version to be published; have agreed on the journal to which the article has been submitted; and agree to be accountable for all aspects of the work.

## Consent

The research was conducted ethically in accordance with the World Medical Association Declaration of Helsinki. The patients have given their written informed consent on admission to use their prospective data base and files for research work.

## Provenance and peer review

Our study was non-commissioned and externally peer-reviewed.

## Registration of research studies

This research has been registered with the Research Registry number: 7361.

## Guarantor

Warsinggih

## Declaration of competing interest

The authors declare that they have no conflict of interests.
